# The targeted anti-*Salmonella* bacteriophage attenuated the inflammatory response of laying hens challenged with *Salmonella Gallinarum*

**DOI:** 10.1016/j.psj.2022.102296

**Published:** 2022-10-29

**Authors:** Abdolreza Hosseindoust, SangHun Ha, Anushka Lokhande, JunYoung Mun, Young In Kim, JinSoo Kim

**Affiliations:** ⁎ Department of Animal Industry Convergence, Kangwon National University, Chuncheon, 24341, Republic of Korea; † CTC Bio, Inc., Seoul, 138-858, Republic of Korea

**Keywords:** immunity, cytokine, challenge, liver, fowl typhoid

## Abstract

Fowl typhoid is a severe disease caused by *Salmonella Gallinarum* with considerable mortality and morbidity in laying hen farms. The current study has focused on controlling the infection in laying hens using anti-*Salmonella* spp. bacteriophage. The treatments included, PC, without challenge; NC, *S. Gallinarum* challenged (**SGC**); B5, 5 mg bacteriophage/kg + SGC; B10, 10 mg bacteriophage/kg + SGC. The *Salmonella* shedding, inflammatory responses, and gene expression of pro-inflammatory cytokines, toll-like receptor (**TLR**), and heat shock protein (**HSP**) in the jejunum, liver, and thigh muscle were tested in laying hens. Supplementation of bacteriophage reduced the abundance of *S. Gallinarum* in the excreta at d 3, 7, and 14. The abundance of *S. Gallinarum* was lower in the B10 than the B5 at d 7. Supplementation of bacteriophage decreased the abundance of *S. Gallinarum* in the oviduct, spleen, and cecum at d 14. The laying hens in the NC group showed an increased relative spleen weight compared with the PC and B10 treatments. Among the SGC treatments, the NC treatment showed higher gene expressions of IL-4 compared with the B5, higher gene expressions of interferon (**IFNγ**), TLR4, and tumor necrosis factor-α (**TNF-α**) compared with the B5 and B10, and higher gene expressions of HSP27 compared with the B10 in the jejunum. Dietary supplementation of B10 decreased the mRNA expressions of TLR4 and TNF-α compared with the B5 treatment in the jejunum. The NC treatment showed the highest gene expressions of HSP27, TLR4, and TNF-α in the liver. Dietary supplementation of B10 showed lower mRNA expressions of HSP27 compared with the B5 treatment in the liver. Moreover, the IFNγ and HSP27 were upregulated in the NC treatment compared with the B5 and B10 in the muscle. In conclusion, it can be suggested that bacteriophage is an effective supplement to control *S. Gallinarum* infection in laying hens and possibly lower horizontal contaminations in laying hen flocks.

## INTRODUCTION

*Salmonella* species are the common inhabitants in the intestine of farm animals (Kogut et al., 2020; Kairmi et al., 2022). Fowl typhoid is a severe disease caused by *Salmonella Gallinarum* with high mortality and morbidity, particularly in brown-shell egg layers (Seo et al., 2019). Outbreaks of *S. Gallinarum* have frequently occurred in laying hen farms with considerable economic losses (Seo et al., 2019). *S. Gallinarum* is a bird-specific pathogen with systemic infection initiating from the intestine and gradually infecting the other organs (Hosseindoust et al., 2018). The laying hen immune response to control *S. Gallinarum* infection is not clearly understood, however, it has already been known that the enterocytes, cecal tonsils, and Peyer's patches are the first target of *S. Gallinarum* (Setta et al., 2012). The inflammatory response initiates in the intestine in response to lipopolysaccharides and flagella from *S. Gallinarum* presence (Gast et al., 2021). In the next phase of infection, the *S. Gallinarum* is transported to other organs through mucosal macrophages or dendritic cells (Sutton et al., 2022). Therefore, it is necessary to control *Salmonella* infection at the first steps in the intestine in order to diminish the consequences. Improving the persistence of birds against *S. Gallinarum* will reduce substantial economic losses by decreasing mortality, morbidity, and transmission.

Improved sanitary conditions, administration of antibiotics and vaccines have been effective ways to control *Salmonella* infection (Setta et al., 2012; Seo et al., 2019; Gast et al., 2021; Lee et al., 2021), however, the bacterial resistance and antibiotic residual in eggs limited the use of antibiotics worldwide. Further, the nonselective specification of antibiotics interferes with intestinal microbiota and eliminates several advantageous bacteria that may increase diarrhea incidence (Kairmi et al., 2022). In this regard, the global interest has been shifted to explore new protective strategies to control the incidence of fowl typhoid in laying hens. However, antibiotics had been used for a long time as part of an effective controlling strategy for *Salmonella* in poultry farms, and the use of nonantibiotic products can greatly reduce the horizontal transmission of *Salmonella* in farms. Horizontal transmission is crucial in the epidemiology of fowl typhoid disease because some infected laying hens turn to be long-term asymptomatic carriers and transfer *Salmonella* to noninfected layers. This is an important issue in breeder farms through transferring *Salmonella* to their progeny by polluted eggs. The use of nonantibiotic factors such as probiotics (Hosseindoust et al., 2018) and bacteriophage (Adhikari et al., 2017; Choi et al., 2020; Lee et al., 2021) have shown preventive and therapeutic effects on *Salmonella* infection control through the gastrointestinal tract. The specificity of bacteriophages in omitting the targeted pathogens increases its importance to be used in challenged laying hens. Moreover, the bacteriophages can be used for a longer period before and after the incidence of fowl typhoid in order to prevent the spread of the disease in the beginning stages. With the continuous increase in production technology, semi-pH-stable bacteriophages were obtained. The engineered bacteriophages (**BP**) are able to survive and multiply in the intestinal lumen environment and target *Salmonella* in chickens (Huang et al., 2022), laying hens (Adhikari et al., 2017; Lee et al., 2021), and pigs (Lee et al., 2016; Hosseindoust et al., 2017). Bacteriophages can be administrated in single or mixture forms to mitigate the severity of diarrhea and pathogenic growth in the gastrointestinal tract. This study was designed to evaluate the effects of dietary anti-*S. Gallinarum* bacteriophage on the disease control, immune status, *Salmonella* spp. growth, of laying hens challenged with *S. Gallinarum*.

## MATERIALS AND METHODS

### Animals and Experimental Design

The experiment was approved by the Institutional Animal Care and Use Committee, Kangwon National University (KW-210503-6). A total of 24 (40-wk-old) Hy-Line Brown layers were subjected to be distributed among 4 treatments with 6 replicates in individual wire-layer battery cages. Excreta samples were collected before starting the challenge to confirm laying hens were *S. Gallinarum* negative. The treatments included: Control, without challenge; NC, *S. Gallinarum* challenged (**SGC**); B5, 5 mg bacteriophage/kg + SGC; B10, 10 mg bacteriophage/kg + SGC. The experiment was conducted for 14 d. The diets were formulated to provide all of the nutrients to meet or exceed the nutrient requirements listed in the Hy-Line nutrient specification handbook. After 7-d adaptation, the *S. Gallinarum* strain KVCC BA 0700722 challenge was performed at d 0 by oral gavage with 3.7 × 10^8^ CFU.

### Bacteriophages Preparation

The bacteriophage product was a powder-type feed additive manufactured by mixing excipients with lyophilizate of bacteriophage comprising Salmonella bacteriophages with high antibacterial activity against *S. Gallinarum*. The bacteriophage had been tested to have no side effects on the host animal, obtaining thermostability to 90°C and pH-stability to pH = 3.5. The *S. Gallinarum* was targeted by supplementing bacteriophages in the diet. The sewage and excreta of farm chickens in Korea were used for bacteriophage isolation steps such as plaque isolation, cultivation process with host strains, purification, and filtration. The manufacture of the selected bacteriophage (BF2211) was conducted using a mix of individual bacteriophage powder prepared as recommended by Lee et al. (2021). In brief, the bacteriophage was co-cultivated with log-phase host strain in tryptic soy broth (Detroit, MI) at 37°C until the lysis of host bacterial cells will be observed. The culture was centrifuged at 10,000 *g* for 30 min at 4°C. The resultant supernatant was filtered with a 0.45-m syringe filter. The procedure, from co-cultivation to filtration, was repeated twice to enhance the bacteriophage titer. The obtaining filtrates containing individual bacteriophage were freeze-dried after adding Maltodextrin (50%, w/v) and pulverized using a milling machine. The resultant powder of individual bacteriophage was used for manufacturing the bacteriophage. The bacteriophage was fed to laying hens after mixing it with commercial complete feed at a 0.05% and 0.1% weight ratio. The content of bacteriophage in the bacteriophage product was adjusted to be approximately above 10^8^ plaque-forming units per g (pfu/g) for *S. Gallinarum* species.

### Organ Samples and RNA Extractions

Total RNA was isolated from the Jejunum (50 mg), livers (50 mg), and spleens (100 mg) samples using Trizol reagent (Invitrogen, Carlsbad, CA) according to the manufacturer's instruction (Lee et al., 2021). Extracted RNA was quantified to 1 μg/μl and cDNA synthesis was conducted using the Improm-II Reverse transcription system (Promega, Fitchburg, MA) and PCR was performed using Mx3000P real-time PCR (Stratagen, La Jolla, CA). The results were expressed as a relative expression by using the delta-delta method. The primers of IL-4, IL-6, interferon-γ (**IFNγ**), heat shock protein-27 (**HSP27**), toll-like receptor-4 (**TLR4**), and tumor necrosis factor-α (**TNFα**) were presented in Table 1. In this process, the house-keeping gene, β-actin was introduced to adjust the quantity of input cDNA to maintain the role in internal control (Lee et al., 2021). A total of 20 μL reaction system included 10 μL SYBR Premix Ex Taq, 0.8 μL of forward and reverse primer (10 μM), 0.4 μL ROX Reference Dye II (50 ×), 2.0 μL cDNA template, and 6 μL dd H_2_O. Cycling conditions were as followed: 30 s at 95°C, 40 cycles of denaturation step at 95°C for 3 s, 60°C annealing step for 34 s, and a 72°C extension step for 15 s.

**Table 1:** Genes and primer sequences used for real time PCR.

Gene	Primer sequence (5´ to 3´)
β actin_F	CAACACAGTGCTGTCTGGTGGTA
β actin_R	ATCGTACTCCTGCTTGCTGATCC
IL4_F	TGTGCCCACGCTGTGCTTACA
IL4_R	CTTGTGGCAGTGCTGGCTCTCC
IL6_F	AGAAATCCCTCCTCGCCAAT
IL6_R	AAATAGCGAACGGCCCTCA
IFNγ_F	CTGAAGAACTGGACAGAGAG
IFNγ_R	CACCAGCTTCTGTAAGATGC
HSP27_F	GGAGATCACCGGCAAACACG
HSP27_R	CCTCCACTGTCAGCATCCCA
TLR4_F	GTCTCTCCTTCCTTACCTGCTGTTC
TLR4_R	AGGAGGAGAAAGACAGGGTAGGTG

Abbreviations: HSP, heat shock protein; IL, interleukin; IFN, Interferon; TLR, toll like receptor; TNF, tumor necrosis factor.

### Salmonella Enumeration

Excreta samples were taken on d 7 and 14, and cecum samples were collected on d 14 from all birds to enumerate *Salmonella* spp. 1 g of sample were collected and diluted 10‐fold (1:9, w/v), by blending them with anaerobically sterilized peptone solution and diluted (Lee et al., 2021). Afterward, a 0.1 mL sample serially diluted by 10^3^ to 10^7^ was spread onto sterilized flat Xylose lysine deoxycholate agar (Difco Laboratories, Franklin Lakes, NJ) in an incubator at 37°C for 24 h, followed by the manual provided by Difco, and expressed as log CFU/g. After slaughtering birds, the contents of the cecum and excreta, spleen, and oviduct were moved into sterilized plastic tubes and directly stored in liquid nitrogen. And all samples were stored at –80°C before analysis. Approximately 2 g of each sample was homogenized on the sterilized mortar by pouring liquid nitrogen into the mortar. Then approximately 250 mg of the frozen sample powders were used for genomic DNA extraction. Genomic DNA extraction was conducted using QIAamp DNA stool mini kit (Qiagen, Hilden, Germany). The reaction mixture was comprised of 1 uL of templet DNA, 10 uL of Brilliant II SYBR Green QPCR Master Mix (Agilent, Santa Clara, CA), 0.4 uL of each primer, and tertiary distilled water to a final volume of 20 mL, then PCR was conducted by using Mx3000P real-time PCR (Stratagen). The condition of PCR and the primers of both total bacteria and *S. Gallinarum* were constructed according to the method of Lee et al., 2013. The primers of total bacteria and *S. Gallinarum* was F-5′-GTATGGTTATTAGACGTTGTT-3′, R-5′-TATTCACGAATTGAATACTC-3′. The detected Ct values of *S. Gallinarum* were normalized by Ct values of total bacteria, then the results were expressed by using the delta-delta Ct method following the method described by Hosseindoust et al. (2020).

### Organ Weight

At the age of 42 wk, all birds were euthanized by cervical dislocation. The liver and spleen were collected to calculate the relative organ weight (percentage of BW).

### Statistical Analysis

Statistical analyses were processed based on a completely randomized design by the GLM procedure of SAS (SAS Inst. Inc., Cary, NC). Laying hen was considered as an experimental unit for all parameters. Factor analysis of variance was based on Tukey multiple range tests at *P* < 0.05 statistical level.

## RESULTS

### Intestine and Organ Microflora

The *Salmonella* spp. population in the excreta and cecum digesta of laying hens is shown in Table 2. The laying hens in the NC group exhibited the highest population of *Salmonella* spp. in the excreta (*P* < 0.05) at d 7. The B5 and B10 treatments showed a higher population of *Salmonella* spp. compared with the PC (*P* < 0.05). At d 14, SGC treatments showed a higher cecal and excreta *Salmonella* spp. population compared with the laying hens in the PC treatment. The abundance of *S. Gallinarum* was evaluated in the excreta, oviduct, spleen, and cecum (Table 3). The PC treatment was S. *Gallinarum*-negative throughout the experiment. Supplementation of bacteriophage reduced (*P* < 0.01) the abundance of *S. Gallinarum* in the excreta at d 3, 7, and 14. The abundance of *S. Gallinarum* was lower in the B10 than the B5 at d 7. Supplementation of bacteriophage decreased (*P* < 0.01) the abundance in the oviduct, spleen, and cecum at d 14.

**Table 2:** *Salmonella* spp. count (log CFU/g) in cecum and feces of laying hens.

Treatments	PC	NC	B5	B10	SEM	*P*-value
SGC	-	+	+	+		
BP (%)	0	0	0.05	0.1		
7 d						
Excreta	1.15^c^	4.43^a^	3.60^b^	3.52^b^	0.15	<0.001
14 d						
Cecum	1.77^b^	3.19^a^	2.76^a^	2.66^a^	0.15	<0.001
Excreta	1.13^b^	2.98^a^	2.51^a^	2.45^a^	0.19	<0.001

Abbreviations: BP, bacteriophage; SGC, *Salmonella Gallinarum* challenge.

a,b Means values with different superscripts with same row differ (*P* < 0.05).

**Table 3:** *Salmonella Gallinarum* abundance in excreta, oviduct, spleen, and cecum of laying hens.

Treatments	NC	PC	B5	B10	SEM	*P*-value
SGC	-	+	+	+		
BP (%)	0	0	0.05	0.1		
3 d						
Excreta	-	1.00^a^	0.54^b^	0.39^b^	0.10	<0.001
7 d						
Excreta	-	1.00^a^	0.36^b^	0.17^c^	0.07	<0.001
14 d						
Excreta	-	1.00^a^	0.23^b^	0.19^b^	0.14	<0.001
Oviduct	-	1.00^a^	0.30^b^	0.27^b^	0.15	0.005
Spleen	-	1.00^a^	0.36^b^	0.24^b^	0.11	0.002
Cecum	-	1.00^a^	0.06^b^	0.09^b^	0.12	<0.001

Abbreviations: BP, bacteriophage; SGC, *Salmonella Gallinarum* challenge.

a∼c Means values with different superscripts with same row differ (*P* < 0.05).

### Immune Organ Size

As shown in Table 4, when the *S. Gallinarum-*challenged laying hens in the PC treatment showed a higher relative weight of liver, however, there was no difference between bacteriophage treatments. The laying hens in the NC group showed an increased relative spleen weight compared with the PC and B10 treatments.

**Table 4:** Relative weights of liver and spleen (g/ 100g BW) of laying hen.

Treatments	PC	NC	B5	B10	SEM	*P*-value
SGC	-	+	+	+		
BP (%)	0	0	0.05	0.1		
Liver	2.20^b^	3.02^a^	2.37^b^	2.48^b^	0.10	0.044
Spleen	0.09^c^	0.16^a^	0.15^ab^	0.11^bc^	0.01	0.001

Abbreviations: BP, bacteriophage; SGC, *Salmonella Gallinarum* challenge.

a,b Means values with different superscripts with same row differ (*P* < 0.05).

### Gene Expression in Jejunum

**Figure 1:**
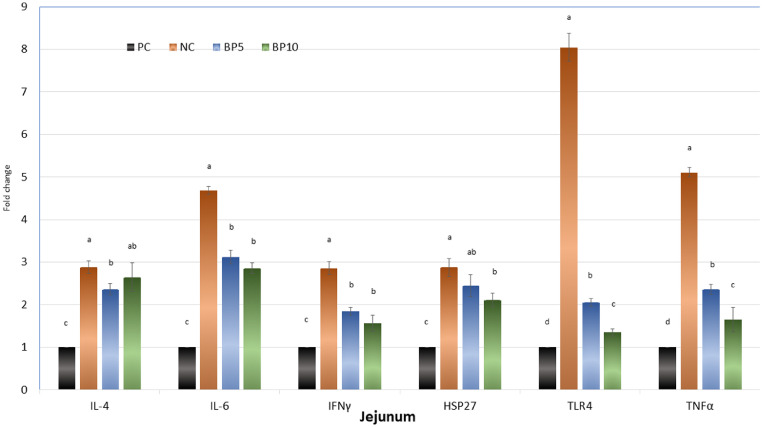
Relative expression of Interleukin 4 (IL-4), interleukin-6 (IL-6), interferon (IFNγ), heat shock protein-27 (HSP27), toll-like receptor-4 (TLR4), and tumor necrosis factor-α (TNFα) in the jejunum of laying hens. Abbreviations: PC, without challenge; NC, S. Gallinarum challenged (SGC); B5, 5 mg bacteriophage/kg + SGC; B10, 10 mg bacteriophage/kg + SGC. Error bars represent standard error of means. Bars with different letters (A–D) differ significantly across all 4 treatment groups (*P* < 0.05).

The PC treatment showed the lowest mRNA expressions of IL-4, IL-6, IFNγ, HSP27, TLR4, and TNF-α in the jejunum compared with the other treatments (*P* < 0.05, Figure 1). Among the SGC treatments, the NC treatment showed higher gene expressions of IL-4 compared with the B5, higher gene expressions of IFNγ, TLR4, and TNF-α compared with the B5 and B10, and higher gene expressions of HSP27 compared with the B10. Dietary supplementation of B10 decreased the mRNA expressions of TLR4, and TNF-α compared with the B5 treatment (*P* < 0.05). There were no differences in gene expression of IL-4, IL-6, IFNγ, and HSP27 between the B5 and B10.

### Gene Expression in Liver

**Figure 2:**
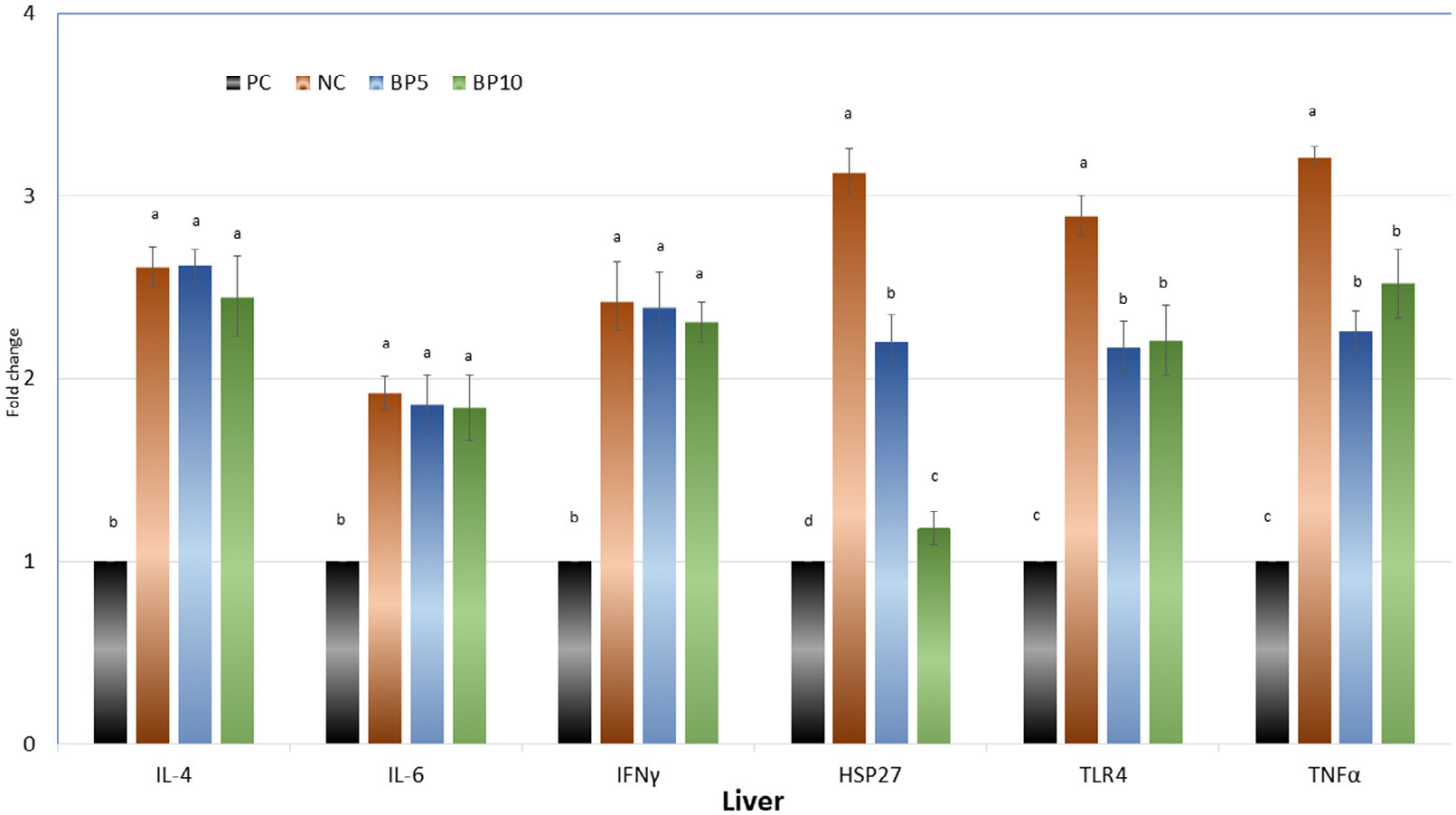
Relative expression of Interleukin 4 (IL-4), interleukin-6 (IL-6), interferon (IFNγ), heat shock protein-27 (HSP27), toll-like receptor-4 (TLR4), and tumor necrosis factor-α (TNFα) in the liver of laying hens. Abbreviations: PC, without challenge; NC, *S. Gallinarum* challenged (SGC); B5, 5 mg bacteriophage/kg + SGC; B10, 10 mg bacteriophage/kg + SGC. Error bars represent standard error of means. Bars with different letters (A–D) differ significantly across all 4 treatment groups (*P* < 0.05).

The PC treatment showed the lowest mRNA expressions of IL-4, IL-6, IFNγ, HSP27, TLR4, and TNF-α in the jejunum compared with the other treatments (*P* < 0.01, Figure 2). Among the SGC treatments, there was no difference in IL-4, IL-6, and IFNγ gene expression, however, the NC treatment showed higher gene expressions of HSP27, TLR4, and TNF-α compared with the B5 and B10. Dietary supplementation of B10 showed lower mRNA expressions of HSP27 compared with the B5 treatment (*P* < 0.05), however, no difference was observed in gene expression of TLR4 and TNF-α.

### Gene Expression in Muscle

**Figure 3:**
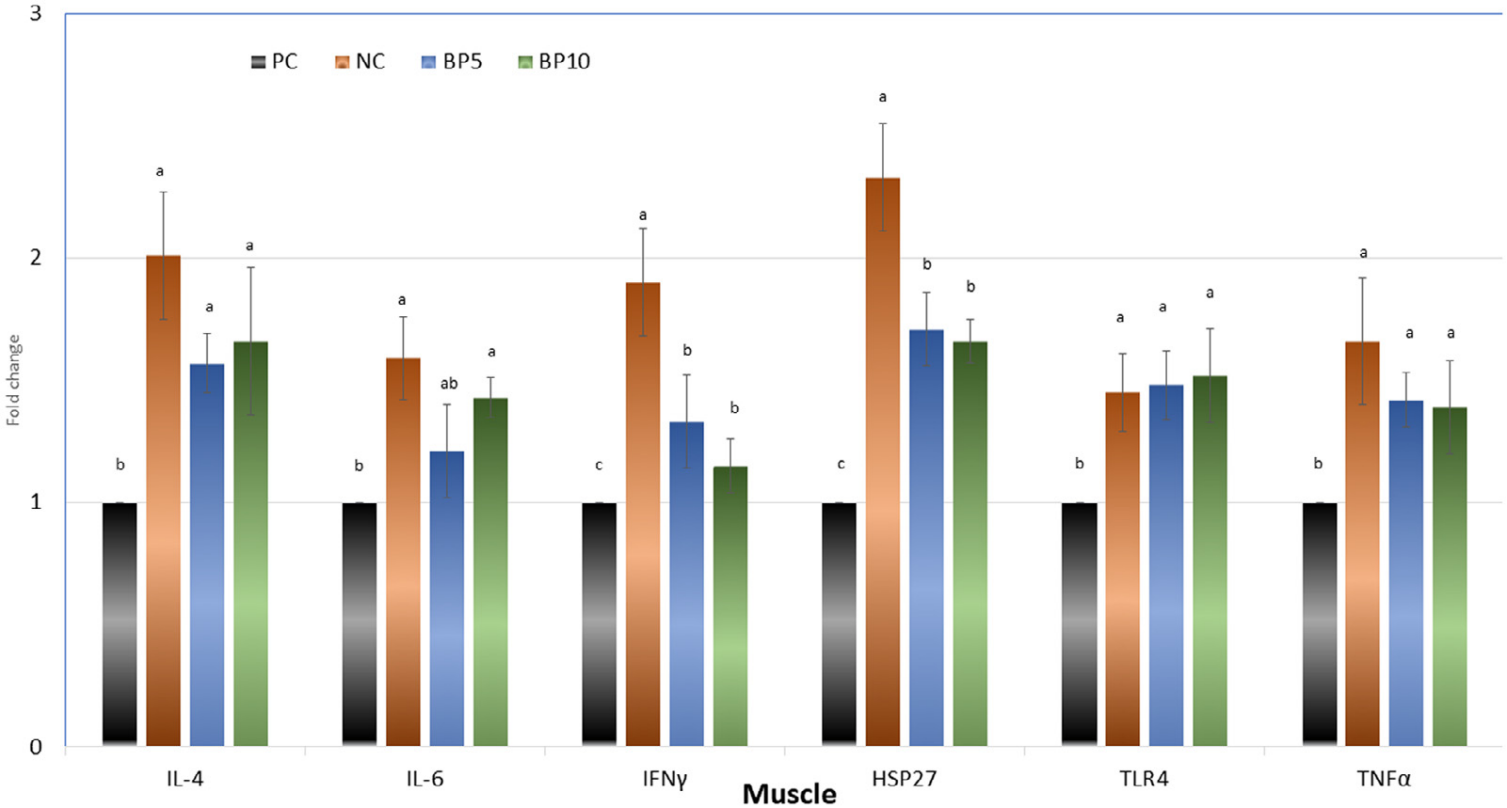
Relative expression of Interleukin 4 (IL-4), interleukin-6 (IL-6), interferon (IFNγ), heat shock protein-27 (HSP27), toll-like receptor-4 (TLR4), and tumor necrosis factor-α (TNFα) in the thigh muscle of laying hens. Abbreviations: PC, without challenge; NC, *S. Gallinarum* challenged (SGC); B5, 5 mg bacteriophage/kg + SGC; B10, 10 mg bacteriophage/kg + SGC. Error bars represent standard error of means. Bars with different letters (A–D) differ significantly across all 4 treatment groups (*P* < 0.05).

The PC treatment showed the lowest mRNA expressions of IL-4, IL-6, IFNγ, HSP27, TLR4, and TNF-α in the jejunum compared with the treatments (*P* < 0.01, Figure 3). Among the challenged laying hens, the gene expression of IL6 was upregulated in the NC treatment compared with the B5. Moreover, the IFNγ and HSP27 were upregulated in the NC treatment compared with the B5 and B10, however, the mRNA gene expressions of TLR4 and TNF-α were not affected.

## DISCUSSION

The intestine is an important organ with considerable effects on the immune system through intestinal microbiota (Hosseindoust et al., 2020; Kogut et al., 2020; Huang et al., 2022). The reduction of *Salmonella* spp*.* counts in phase 1 compared with phase 2 may be due to the immediate effects of bacteriophage. It has been suggested that bacteriophages mostly have delayed antipathogenic effects and show a better performance over a long supplementation period (Lee et al., 2021). The lack of difference in counts of *Salmonella* spp. in the excreta and cecum between different doses of bacteriophages a d 14 may show the efficiency of bacteriophage supplementation even at the lower dose in the second phase. More specifically, our findings demonstrate that the dramatic decrease in the count of *S. Gallinarum* in the excreta can decrease the horizontal transfer of *S. Gallinarum* among the hens. The control of fowl typhoid disease is according to the elimination of infected birds from the flock, mainly due to removing the cross-infection through excreta.

Among organs, the intestine, liver, and spleen were known to be potential foci for *S. Gallinarum* proliferation (Da Silva et al., 2016; Zhou et al., 2020). In agreement, the reduction of relative abundance of *S. Gallinarum* in the oviduct, spleen, and cecum was obtained with bacteriophage supplementation in the challenged laying hens. An insignificant difference between bacteriophage treatments may be associated with the fact that laying hens require lower doses of bacteriophage in the second stage of the disease. After penetration of *S. Gallinarum* from intestinal cells to the circulation, they penetrate to systemic sites such as the spleen and liver (Da Silva et al., 2016). The multiplication *S. Gallinarum* in internal organs stimulates the immune system and increases inflammation. It is difficult to explain how such a reduction in the population of *S. Gallinarum* can be seen in internal organs such as the liver and spleen, but there might be 2 reasons to explain this effect. First, the role of bacteriophage in the elimination of *S. Gallinarum* in the intestine, as the main site of proliferation, may result in the reduction of inflammatory responses and aiding the immune system of laying hens in order to eliminate *S. Gallinarum* in systemic organs. Second, however, the count of bacteriophages in the organs was not performed in the current study, bacteriophages can penetrate into *Salmonella* bacterium and use them as a carrier to be transferred to systemic organs and eliminate the targeted pathogens.

The pathogenic pathogen-associated molecular in the intestine influence TLRs to initiate activation of nuclear factor-κB in macrophages and subsequent inflammatory responses (Kamimura et al., 2017; Sutton et al., 2022; Tong et al., 2022). The TLR stimulation increase production of pro-inflammatory cytokines such as TNF-α, IL-1β, and IL-6 (Al-Zghoul and Mohammad Saleh, 2020). *Salmonella*, gram-negative bacteria, produces LPS, which stimulates TLR4 as the most sensitive TLR to pathogenic LPS. The TLR4 expression was reduced in the jejunum and liver of the bacteriophage-fed laying hens compared to the NC laying hens. This means that the signaling was highly dependent on *Salmonella* infection and the paracellular presence of *S. Gallinarum* in the intestine. There was no difference in TLR4 gene expression between B5 and B10 in the liver and jejunum, however, the B10 showed a downregulated TLR4 gene expression in the jejunum compared with the B5 treatment. The current achievement pinpoints the importance of the higher doses of bacteriophages in the intestine, which possibly reduce the population of *S. Gallinarum* with higher efficiency. The downregulated jejunal TLR4 gene expression can be associated with the reduction of *S. Gallinarum* and TLR stimulators in the intestine. These events accentuate the requirement to supplement the higher doses of bacteriophage in challenging environments.

Bacteriophages are new types of animal antipathogen agents, which increase growth performance and improve animal health (Adhikari et al., 2017; Hosseindoust et al., 2017, 2018; Lee et al., 2021). In the current study, the preventive role of bacteriophage on gene expression of TLR4 and inflammatory cytokines were relatively higher in the jejunum rather than in the liver and muscle. *S. Gallinarum* infection increased the pro-inflammatory cytokine IL4, IL6, TNF-α, and IFN-γ mRNA levels compared with those in the unchallenged control, indicating that *S. Gallinarum* challenge was highly triggered inflammatory responses in the intestine. The dietary bacteriophage mitigated the inflammatory response and contributed to maintaining the immunological changes by reducing the expression of TLR4, IFNγ, and TNF-α in the jejunum and liver of bacteriophage-supplements laying hens. During acute inflammation, the intensity of damage was reduced by cellular and molecular adjustments (Hosseindoust et al., 2022). Inflammatory cytokines are the major mediators in the innate immune response process (Hosseindoust et al., 2022). The immunity reactions initiate by TLR4 stimulation and proinflammatory cytokines production, including IL-1β, IL-6, TNF-α, and IFNγ (Alizadeh et al., 2016). In this study, the expression of TNF-α was downregulated in the liver and jejunum of bacteriophage-supplemented laying hens compared with the NC, showing a lower inflammatory response. The protective effect of bacteriophages on TLR-induced intestinal inflammation can be associated with the reduction of *S. Gallinarum* and their signaling effects.

Lesions of *Salmonella* infection appear in the liver and spleen tissues because of bacteremia, which extensively spreads in the organism (Shao et al., 2013; Da Silva et al., 2016; Choi et al., 2020; Lee et al., 2021). The unique vascular system in the liver indicates the sensitivity to inflammation by intestinal-secreted pathogen-associated molecules, as well as danger-associated molecular patterns in hepatocytes (Lee et al., 2021). The activation of pathogen-associated molecules increases the production of pro-inflammatory cytokines (Xie et al., 2002). Liver is one of the main organs for the synthesis of acute-phase proteins under influence of pro-inflammatory cytokines (Da Silva et al., 2016; Tong et al., 2022). In the current study, the increase in liver and spleen weight can be associated with acute-phase proteins and upregulation of inflammatory cytokines including IL-6, TNF-α, and IFNγ. *S. Gallinarum* increased inflammation in the spleen, which was accompanied by enlargement or splenomegaly. Diagnosis of bacterial infections of hemorrhagic or marble spleen can mainly be observed based on spleen color, size, and gross lesions (Oh et al., 2010). The *Salmonella* challenge was shown by spleen enlargement by 78% higher weight in the NC treatment compared to the PC. Infection, cirrhosis, leukemia, and injury are the main reasons for increasing spleen size (Gent and Blackie, 2017). Hypertrophy is the response of the spleen to intracellular pro-inflammatory cytokine production induced by *Salmonella*, which results in splenic lesions and hyperplasia (Oh et al., 2010; Choi et al., 2020). In agreement, laying hens challenged with *S. Typhimurium* (Oh et al., 2010; Tian et al., 2018) showed a higher relative weight of liver and spleen. In our study, the significant weight change in the liver and spleen of laying hens indicated that the *Salmonella* challenge was effectively performed.

In conclusion, our results showed the supplementation of bacteriophage effectively decreased *S. Galinarum* infection in laying hens with the potential to be administered in the path toward the development of nonantibiotic farming. The elimination of *S. Galinarum* in the excreta shows the potential for reducing cross-infection among laying hens on a farm scale.
